# Therapeutic Dose Response of Acoustic Cluster Therapy in Combination With Irinotecan for the Treatment of Human Colon Cancer in Mice

**DOI:** 10.3389/fphar.2019.01299

**Published:** 2019-11-19

**Authors:** Nigel Bush, Andrew Healey, Anant Shah, Gary Box, Vladimir Kirkin, Spiros Kotopoulis, Svein Kvåle, Per Christian Sontum, Jeffrey Bamber

**Affiliations:** ^1^Joint Department of Physics, Institute of Cancer Research, London, United Kingdom; ^2^CRUK Cancer Therapeutics Unit, The Institute of Cancer Research, London, United Kingdom; ^3^Phoenix Solutions AS, Oslo, Norway

**Keywords:** acoustic cluster therapy, microbubbles, ultrasound, drug delivery, dose/response, irinotecan, colorectal cancer

## Abstract

**Introduction:** Acoustic Cluster Therapy (ACT) comprises coadministration of a formulation containing microbubble-microdroplet clusters (PS101) together with a regular medicinal drug and local ultrasound (US) insonation of the targeted pathological tissue. PS101 is confined to the vascular compartment and when the clusters are exposed to regular diagnostic imaging US fields, the microdroplets undergo a phase shift to produce bubbles with a median diameter of 22 µm. Low frequency, low mechanical index US is then applied to drive oscillations of the deposited ACT bubbles to induce biomechanical effects that locally enhance extravasation, distribution, and uptake of the coadministered drug, significantly increasing its therapeutic efficacy.

**Methods:** The therapeutic efficacy of ACT with irinotecan (60 mg/kg i.p.) was investigated using three treatment sessions given on day 0, 7, and 14 on subcutaneous human colorectal adenocarcinoma xenografts in mice. Treatment was performed with three back-to-back PS101+US administrations per session with PS101 doses ranging from 0.40–2.00 ml PS101/kg body weight (n = 8–15). To induce the phase shift, 45 s of US at 8 MHz at an MI of 0.30 was applied using a diagnostic US system; low frequency exposure consisted of 1 or 5 min at 500 kHz with an MI of 0.20.

**Results:** ACT with irinotecan induced a strong, dose dependent increase in the therapeutic effect (R^2^ = 0.95). When compared to irinotecan alone, at the highest dose investigated, combination treatment induced a reduction in average normalized tumour volume from 14.6 (irinotecan), to 5.4 (ACT with irinotecan, p = 0.002) on day 27. Median survival increased from 34 days (irinotecan) to 54 (ACT with irinotecan, p = 0.002). Additionally, ACT with irinotecan induced an increase in the fraction of complete responders; from 7% to 26%. There was no significant difference in the therapeutic efficacy whether the low frequency US lasted 1 or 5 min. Furthermore, there was no significant difference between the enhancement observed in the efficacy of ACT with irinotecan when PS101+US was administered before or after irinotecan. An increase in early dropouts was observed at higher PS101 doses. Both mean tumour volume (on day 27) and median survival indicate that the PS101 dose response was linear in the range investigated.

## Introduction

A prerequisite for successful therapy with a medicinal drug is that the active substance reaches its target pathology and that toxicity to healthy tissue and nontargeted organs is limited. However, once a drug is administrated systemically, the mononuclear phagocyte system, the vascular endothelium, the disrupted tumour blood flow, the interstitial and osmotic pressure, the tumour stroma, endosomal escape, and drug efflux pumps are a few amongst a multitude of other biological barriers that severely restrict its effective delivery from the vascular compartment into the tissue of the targeted pathology (Nizzero et al., 2018). In effect, for a number of drugs, the current, passive transvascular delivery paradigm is inefficient, and insufficient tumour penetration of therapeutic agents to reach effective local concentrations is often the outcome. In combination with low therapeutic indexes, increasing the dosages is not a viable strategy due to serious and widespread adverse effects, generally severely limiting the clinical utility of a range of potent drugs.

Whereas lack of sufficient extravasation of drug to the targeted pathology is an issue over a range of medicinal therapeutic segments, this is predominant in the field of chemotherapy for cancer treatment. Regular chemotherapeutics and a range of more novel immune therapies induce severe side effects at partially effective doses and typically, these medicinal regimes are not completed because the cancer is eradicated but because the body cannot tolerate more treatment. The outcome is then only palliative benefit or life prolongation instead of a cure (Hui and Bruera, 2016). For hepatic metastases from colon and pancreatic cancer, primary pancreatic cancer and triple negative breast cancer treated with standard of care chemotherapy, this is unfortunately often the case.

In order to resolve this fundamental problem, over the past decades, a wide range of concepts to improve on pathology-specific uptake (targeted drug delivery) have been explored (Devarajan and Jain, 2015). Within oncology, numerous drug carrier concepts, e.g., liposomes, micelles, dendrimers, and nanoparticles, have been employed either to passively make use of the passive enhanced permeability and retention effect (Maeda et al., 2000) or in combination with surface ligands to actively promote accumulation in tumour tissue through biochemical affinity to specifically expressed target groups. While huge resources have been spent on finding functional concepts for targeted drug delivery over the last two decades, and despite promising preclinical results for several of these, there has been very limited transition to drug products and clinical practice. In truth, the objective remains essentially unresolved in current standard of care medicinal therapy.

In recent years, several concepts for ultrasound (US) mediated drug delivery have been investigated, some with quite encouraging results (Tsutsui et al., 2004; Martin and Dayton, 2013; Unga and Hashida, 2014). Most of these concepts explore the use of commercially available US contrast microbubbles injected intravenously. Insonation of the target pathology leads to a variety of biomechanical effects that enhance extravasation and distribution of drug molecules to target tissue (Kooiman et al., 2014; Lentacker et al., 2014). Coinjection of Gemcitabine and Sonovue^®^, with localized US insonation for a hypothesized-enhanced drug uptake and therapeutic effect during treatment of pancreatic cancer (PDAC), has been explored in clinical trials with encouraging results (Dimcevski et al., 2016). A similar approach is being investigated for treatment of Glioblastoma in humans (Carpentier et al., 2016). While these studies have shown great promise, there are still several limitations (van Wamel et al., 2016b) and Acoustic Cluster Therapy (ACT) has been developed as a new therapeutic bubble concept specifically designed to improve on the shortcomings of contrast microbubbles for US-targeted drug delivery (Sontum et al., 2015; Healey et al., 2016). ACT exploits different mechanisms to those induced by contrast microbubbles and addresses important deficiencies of the latter. In brief, ACT comprises of an intravenous injection of microbubble-microdroplet cluster dispersion (PS101) coadministration with a drug, followed by a two-step, local US insonification for (i) activation and (ii) delivery enhancement procedure. US activation (at diagnostic US frequencies and MI > 0.10) induces a liquid-to-gas phase shift of the microdroplet component and the formation of large (∼22 µm median diameter) bubbles, referred to as ACT bubbles. The ACT bubbles are designed to have a size distribution that causes them to lodge in the microvasculature of the tissue in which the activation occurred, forming transient occlusions in these capillaries. The subsequent US enhancement step induces controlled volume oscillations of the ACT bubbles that lead to enhanced local permeability of the vasculature and other localized mechanical effects, allowing for improved extravasation of a coinjected drug and its improved distribution into the tumour tissue extracellular matrix. The ACT bubbles, being 1,000 times larger (by volume) than contrast microbubbles, will induce orders of magnitude greater biomechanical work. Furthermore, being lodged in the vascular compartment until they dissolve, the ACT bubbles are in direct contact with a substantial portion of the endothelial wall (Sontum et al., 2015) over 5–10 min. This also allows for prolonged insonation to induce biomechanical effects using low frequency (e.g., 0.5 MHz) low amplitude (MI < 0.30) US. The concept represents an unprecedented approach to US-targeted drug delivery that may improve greatly the efficacy of, for example, current chemotherapy regimen.

ACT has been explored in combination with a range of drugs for enhancing their efficacy in the treatment of several cancer xenograft models in mice, including Abraxane^®^ (nab-paclitaxel) and paclitaxel for treatment of human prostate cancer (Park, 2016; van Wamel et al., 2016b), paclitaxel for treatment of human pancreatic ductal carcinoma (PDAC) (Kotopoulis et al., 2017) and Doxil^™^ for treatment of triple negative breast cancer (companion paper in this journal issue). In these studies, an impressive increase in the therapeutic efficacy over drug alone is observed when combined with the ACT procedure. To date, however, no study using ACT has explored anything other than a fixed dose of PS101, the effect of the timing and duration of the PS101+US procedure, nor the treatment of colon cancer. In the current paper, we evaluate whether PS101+US is able to enhance the efficacy of a clinically relevant drug, irinotecan, for treatment of human colorectal cancer (CRC) in mice. Furthermore, in order to investigate the relationship between PS101 dose and therapeutic response, we examine the effect level of a wide range of PS101 doses. Finally, the effect of varying the timing and duration of the ACT procedure is investigated.

Colorectal cancer is the third most common cancer worldwide and approximately 30% of patients with CRC will develop liver metastases during the course of their disease. Only about 25% of these are amenable to curative-intent treatment through metastatectomyand have a 10-year survival rate of 26% (Minagawa et al., 2000). For this disease, US-targeted treatment of hepatic metastases with ACT has a range of potential applications including: as a part of a neo-adjuvant regime prior to resection to improve survival outcome, to downstage and increase the fraction of patients amenable for curative resection and, finally, to improve on survival outcome and palliation for nonresectable conditions.

## Materials and Methods

### Mice and Tumours

SW620 human colon carcinoma cells (American Type Culture Collection, Manassas, VA, USA, lot no. 8924081) were grown in DMEM containing 10% foetal bovine serum in a humidified atmosphere of 5% CO_2_ at 37°C and passaged before renewal from frozen. Cells were regularly screened for mycoplasma by PCR using in-house primers.

Human tumour SW620 xenografts were established in 6-week-old female athymic nude mice, ICR : Ncr-Foxn1 (nu), bred in-house. Mice were housed in groups of five in individually ventilated cages (IVCs) and allowed access to food and water ad libitum. All mice were treated in accordance with local and national animal welfare guidelines (Workman et al., 2010). The studies were performed under a UK Home Office project license and approved by the Local Animal Welfare & Ethical Review Body.

Before tumour implantation, mice were anaesthetised with isoflurane; a 100µl tumour cell suspension containing 3x10^6^ cells was then slowly injected subcutaneously into the left flank of the recipient mice. Tumours were allowed to grow for 7–14 days and treatment started when the tumours were palpable and had attained an average volume of 90 ± 3mm^3^. Prior to each treatment anaesthesia was induced by subcutaneous (s.c.) injection of Fentanyl citrate: Fluanisone (HypnormVetaPharma Ltd, Leeds, UK) and Midazolam (Hypnovel^®^, Roche Products Ltd, Welwyn Garden City, UK) (0.28:10:4.5mg/kg). During treatments, the mice were maintained on a mouse handling table (Vevo^™^, Fujifilm Visualsonics Inc., Toronto), and the body temperature was controlled thermostatically, with vital signs carefully monitored. Following treatments, mice were kept in a temperature-controlled recovery chamber until fully recovered.

### Therapeutics

Clinical grade irinotecan (CPT-11, Seacross Pharmaceuticals Ltd, UK) was resuspended in 0.9% saline and administered intraperitoneally (i.p.) on days 0, 7, and 14 at a single dose of 60 mg/kg. The first injection of three PS101 doses was injected intravenously (i.v.) approximately 11 min after the irinotecan injection, or irinotecan was injected immediately after the last of three PS101 injections and US insonation.

As ACT treatments employ an anaesthesia step and such are expected to increase animal physical stress (Gargiulo et al., 2012) resulting in a higher systemic sensitivity to irinotecan, an irinotecan doses of 60 mg/kg were chosen; equivalent to 60% of the maximum tolerated dose in literature (Motwani et al., 2001). Literature values show that irinotecan has a maximum blood plasma concentration (C_max_) between 0.5 and 1 h after i.p. injection, at which time, it is in the range of 6–10µg/ml (Araki et al., 1993; Guichard et al., 1998). The irinotecan was cleared to less than 1% of C_max_ within 24 h.

PS101 (Sontum et al., 2015) was provided by Phoenix Solutions AS, (Oslo, Norway). PS101 was prepared by reconstituting commercially available microbubbles, Sonazoid^™^(GE Healthcare), with a microdroplet emulsion of perfluoromethylcylopentane (F2 Chemicals Ltd., UK) microdroplets. The reconstituted PS101 formulation consists of a suspension of small microbubble-microdroplet conjugates (“clusters”) 6 × 10^7^ clusters/ml, with a median cluster diameter of 5 µm. The content of perfluoromethylcylopentane in PS101 is 6.8 mg/ml. For administration of low doses, to allow for acceptable injection volumes, PS101 was diluted in 0.9% saline prior to administration.

### Apparatus for *in Vivo* US Activation and Delivery Enhancement

**Figure 1:**
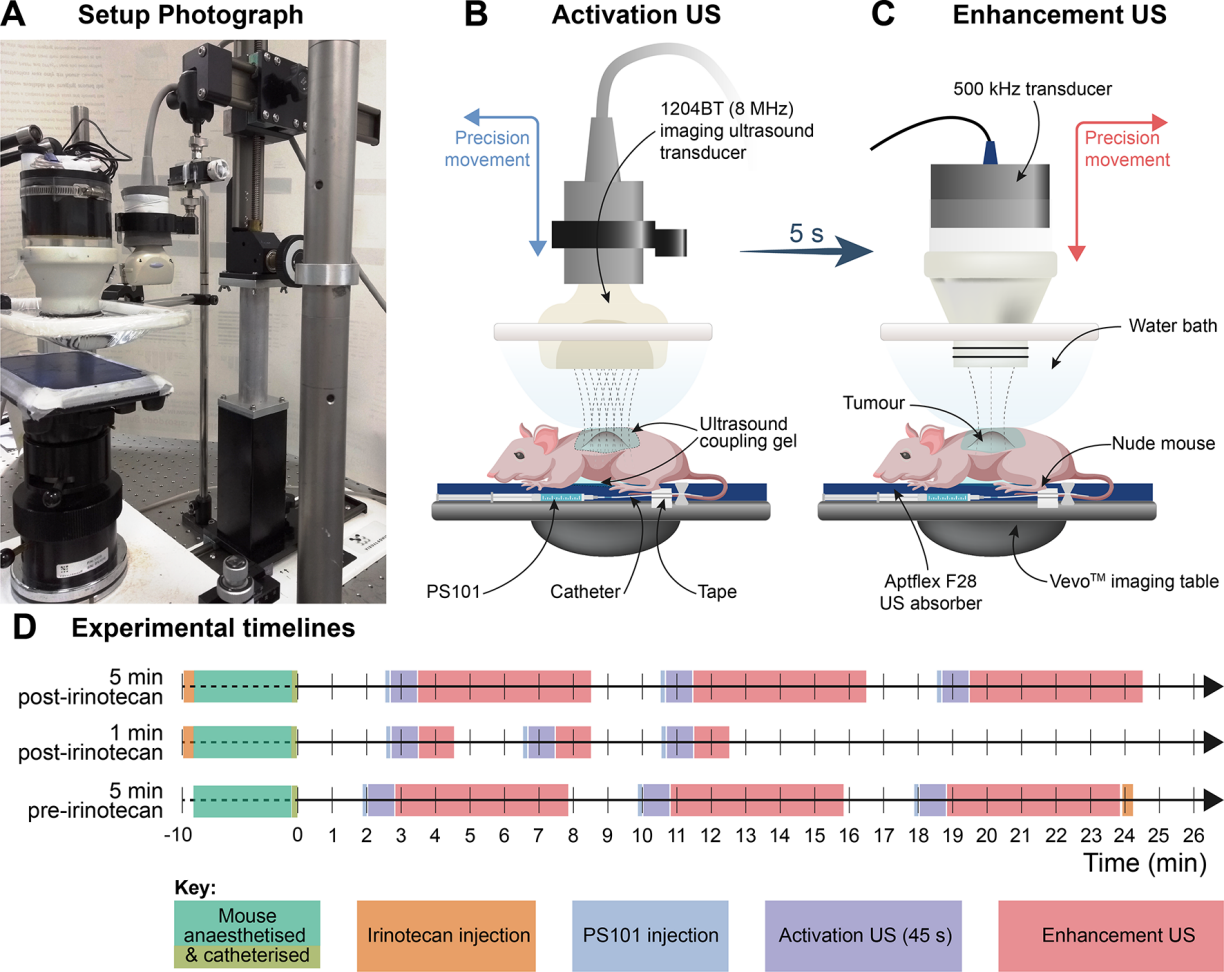
Ultrasound (US) administration apparatus and experimental timelines. (Panel **A**) is a photograph of the activation and delivery enhancement setup showing the animal bed with the low frequency transducer in position for delivery enhancement and the clinical transducer to one side which had been transposed with the other transducer after the activation step; (Panels **B** and **C**) are schematics illustrating the positioning of the activation and delivery enhancement transducers, respectively. The water bath and acoustic transmission gel interface permit easy US transducer-to-tumour depth setting and transducer interposition. The tail vein catheter and tail are tapped to the imaging table. (Panel **D**) shows the timelines for the procedures.

The experimental setup (**Figure 1**) consists of a mouse table covered with a 5-mm thick layer of acoustic absorber (Aptflex F48^™^, Precision Acoustics, Dorchester, UK) which reduces acoustic reflections from the far side of the animal. An open polyethylene water bath mounted above the animal and two US transducers for separate agent activation and delivery. US activation of PS101 and simultaneous imaging confirmation of activation was achieved with a clinical, diagnostic Aplio XG US scanner (Toshiba Medical Systems Corporation, Tochigi, Japan) combined with a 1204BT linear array to provide simultaneous interleaved, nonlinear contrast mode, and fundamental mode imaging at 8 MHz (van Wamel et al., 2016a). ACT delivery enhancement was achieved separately using a custom made 500 kHz, 55 mm active diameter, single element spherically focused transducer which had a radius of curvature of 90 mm (Imasonic SAS, Voray-sur-l’Ognon, France).

The two transducers were arranged to be interposable on a precision sliding arm (**Figure 1**) mounted above the animal, directed at the subcutaneous tumour, insonating through an open polyethylene water bag above the animal, with acoustic coupling gel between the water bath and the mouse. The tumour centre was positioned to sit at the (single) transmit focus of the activation transducer and beyond the focus of the enhancement transducer (14 cm from its front face). For calculation of delivery enhancement MI values, the enhancement field was characterised by measuring the spatial and temporal peak-negative pressure *a priori in situ* at the equivalent tumour location in the therapy rig, using a calibrated Onda HGL-0200 hydrophone (Onda Corp., Sunnyvale, CA). The activation MI values were given as the output displayed on the Toshiba Aplio US activation scanner.

The anaesthetised mice were positioned prone on the treatment table with their left flank and tumour uppermost. For PS101 delivery, a new catheter was made up prior to each treatment by combining a winged infusion set, Surflo^®^, 27G butterfly needle (Terumo Europe, Leuven, Belgium), 70-mm polyethylene tubing, 0.4-mm internal diameter (Biochrom Ltd, Cambridge,UK), and a 27G, 0.5” needle. The catheter primed with a 0.9% saline solution was inserted into the lateral tail vein of the mouse and patency checked by injecting a small volume (<5 µl) of saline solution. The hub of the cannula was then filled with 0.9% saline and closed with a cap and taped to the animal’s tail with surgical tape, which gave a dead space of 10 µl to be accounted for in subsequent injection. PS101 was drawn into a 1-ml syringe and 60 µl (50 µl effective dose, plus 10 µl to allow for dead space) was injected intravenously into the animal’s lateral tail vein *via* the preplaced catheter. Following the PS101 injection, the tumour was insonated using the 1204BT transducer for 45 s at 8 MHz at an MI of 0.30 for activation, then the US transducers were transposed and the tumour was further insonated for 1 or 5 min (c.f. treatment groups, **Table 1**) at 500 kHz (2 cycle excitation, 125 ms burst period) at an MI of 0.20 for delivery enhancement by excitation of ACT bubble oscillations. PS101 dosing followed by US activation and enhancement was then repeated two more times with the shortest possible time (∼2 min) between the end of an enhancement step and the beginning of the next PS101 injection.

**Table 1:** Summary of the treatment groups; number of mice, PS101 dose, PS101+US/irinotecan order, and enhancement ultrasound duration.

Group	Number of animals	Treatment		PS101+US procedure	
		Drug [i.p.](60 mg/kg)	PS101 Dose [i.v.] 3 x (ml/kg)	Pre-irinotecan or Post-irinotecan	Enhancement US duration (min)
1	8	Saline	–	–	–
2	15	Irinotecan	–	–	–
3	8	Irinotecan	0.40	Post	5
4	10	Irinotecan	1.03	Post	5
5	8	Irinotecan	1.53	Post	1
6	8	Irinotecan	2.00	Post	5
7	9	Irinotecan	2.00	Post	1
8	9	Irinotecan	2.00	Pre	5

### Tumour Treatment Regimes

Animals were randomized into eight treatment cohorts of 8 to 15 mice per group. Two control groups, saline only and irinotecan only, received sham US exposure to mimic additional procedure induced stress on the animals. The six remaining groups received US exposure as described in Apparatus for *in-vivo* ultrasound activation and delivery enhancement section and PS101 at doses from 0.4 to 2.0 ml PS101/kg at various timepoints relative to the irinotecan injection and with various US enhancement durations (**Table 1**).

Groups 2–7 provided results for the condensed dose-response study.

### Liver Toxicity

A liver toxicity study was performed to determine if ACT with or without irinotecan induced any sustained liver damage over a 24-h period. **Table 2** summarizes the treatment groups evaluated. Each group consisted of four mice. All mice were healthy and tumour free. All groups except the irinotecan groups (Groups 3 and 4 **Table 2**) underwent 3 × 45 s activation and 5-min enhancement US directed to treat the entire liver. In Groups 7 and 8 (**Table 2**), the irinotecan was injected i.p. a minimum of 10 min before PS101 injection and US exposure, i.e., mimicking Group 6 from **Table 1**.

**Table 2:** Summary of the liver toxicity study groups.

Group	Number of animals	Treatment (dose)	Blood collection time (h)
1	4	Sonazoid (2.00 ml/kg) + US	1.5
2			24.5
3		Irinotecan (60 mg/kg)	1.5
4			24.5
5		PS101 (2.00 ml/kg) + US	1.5
6			24.5
7		PS101 (2.00 ml/kg) + Irinotecan + US	1.5
8			24.5

#### Blood Collection and Processing

Blood samples were collected 1.5 or 24.5 h after the first treatment and were collected by terminally anaesthetising the mice using isofluraneand performing a cardiac puncture with a 21G needle (Becton Dickinson, Franklin Lakes, NJ, USA) and a heparinised syringe. A volume of approximately 600 µl was collected from each mouse into a 1-ml microcentrifuge tube which had been washed with heparin (Eppendorf, Hamburg, Germany). To separate the serum, the samples were centrifuged at 3,000 rpm at 4°C for 15 min and 300 µl of the supernatant was subsequently transferred to a 1-ml microcentrifuge tube (Eppendorf). Sampled were stored at -20°C until analyzed.

Two liver enzymes, alanine aminotransferase (ALT) and aspartate aminotransferase (AST), were quantified using a UniCelDxC 600 Synchron Clinical System (Beckman Coulter, Brea, CA, USA) following the manufactures recommend procedures and reagents.

Literature values for the normal range in mice were used to minimize the number of animals used. Specifically, the normal range for AST and ALT in mice are reported to be in the range of 300 ± 100 units/L and 100 ± 50 units/L (Oršolić et al., 2010; Gao et al., 2014).

### Monitoring Therapeutic Response

Animals were monitored daily for body weight and tumour size *via* calliper measurement for 120 days after study start. Tumour volumes were calculated using the ellipsoid equation: $\frac{4}{3}\pi {\left(\frac{a}{4}+\frac{b}{4}\right)}^{3}$ Tumour size is reported as fold-increase relative to the size on the day of the first treatment. Body weight was used as a proxy for toxicity. Tumour growth inhibition (TGI) percentage was calculated using the equation: $\frac{\left(V_{c}-V_{t}\right)}{\left(V_{c}-V_{0}\right)}\times 100$ where *V* *_c_* and *V* *_t_* are the mean fold increase of the control and treated tumour respectively, on day 27. *V* *_0_* is the control tumour fold growth at the start of the treatment, which is always equal to 1.

Following the 3Rs of ethical research and current EU directives (Directive 2010/63/EU, 2010), a drug + US-only group was not included in the study as the US exposure levels are well below that which might cause bioeffects (Miller et al., 2008; Nelson et al., 2009). Similarly, groups where the treatment was not expected to affect tumour growth, based on previous publications and literature, were not included and considered outside the scope of this study such as PS101 alone and US alone.

As all animals were sacrificed after 120 days to minimize the unnecessary burden. Mice that survived 120 days with no palpable evidence of tumours and were able to go the three weeks of therapy plus 2-week recovery period were considered complete responders.

### Statistical Analysis

Results for average tumour normalized volume are expressed as mean ± standard error. Statistical comparisons of mouse weights, tumour normalized volume, and liver enzymes were performed using an ordinary one-way ANOVA with multiple comparisons and a two-stage setup method for controlling the false discovery rate, or a student’s t-test where only two groups were compared. Survival was compared using a log-rank (Mantel-Cox) test between two groups. Mice that were unable to complete the 3-week treatment plus 2-week recovery period, are reported as censored subjects as tick marks in the survival curves. All mice are reported in the tumour normalized volume data. Correlation was evaluated using a one tailed, nonparametric, Spearman test. Complete responders were evaluated using a contingency table and a two-sided Fisher’s exact test. A p-value less than or equal to 0.05 was considered statistically significant. All statistical analyses were performed in Prism 8.1.2 (GraphPad Software Inc, San Diego, CA, USA).

## Results

### Toxicity

**Figure 2:**
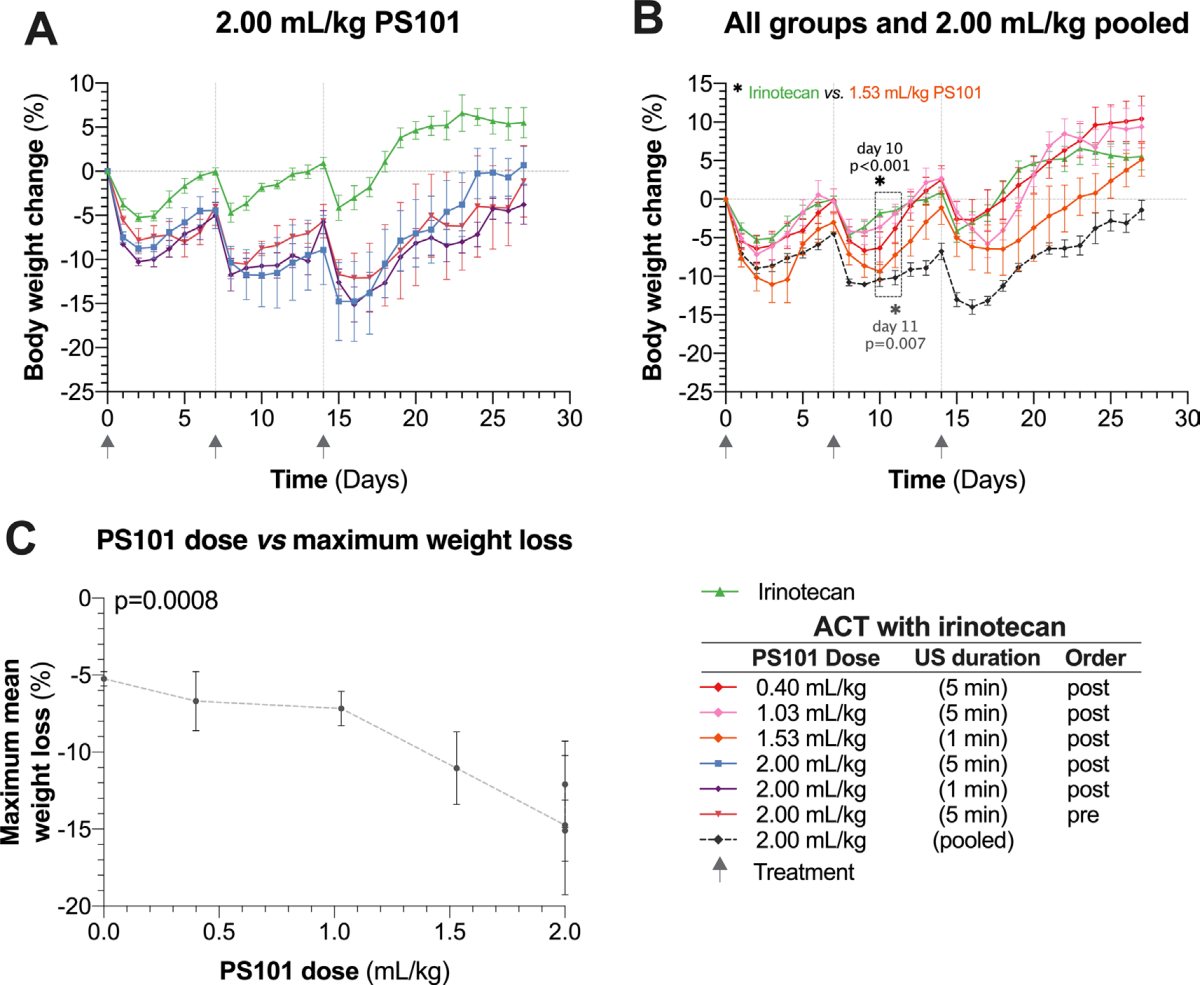
Normalized mouse body weight as a function of time. (Panel **A**) shows the results for irinotecan alone and ACT with irinotecan for the highest dose of PS101 (2.00 ml/kg). Mouse body weight was significantly lower in all the 2.00 ml/kg PS101 groups for the entire 27 days (p = 0.035, ANOVA). (Panel **B**) pools the 2.00 ml/kg groups and compares the weight change against all other ACT with irinotecan groups. Increasing the PS101 dose increased the loss in body weight. The three grey arrows on the time axis indicate the treatment days. (Panel **C**) compares the maximum body weight loss to the PS101 dose. The correlation was significant (p = 0.0008).

Mean body weight changes as a function of time are shown in **Figure 2**. Irinotecan itself was associated with a body weight drop of 5% observed one to two days after each treatment cycle. The mice were able to recover to normal body weight by the day of each subsequent treatment. In contrast, mice treated with the highest PS101 dose (2.00 ml/kg) showed an increase in toxicity observed as a 15% drop in body weight one to two days after treatment. When comparing to the irinotecan group alone, this weight loss was statistically significant for the entire measured period of 27 days (p = 0.035).

The mice treated using ACT with irinotecan (PS101 dose of 2.00 ml/kg) was not able to return to their normal body weight prior to the next treatment cycle. After the three treatment cycles, all mice treated using ACT with irinotecan showed progressive recovery of body weight and the majority of mice reached their starting weight two weeks (the recovery period) after the last treatment. The mean body weight of any 2.00 ml/kg PS101 group never fell below 20% of the starting weight (**Figure 2A**).

This transient increased weight loss following ACT treatment was observed for all PS101 doses (**Figure 2B**). At the lowest PS101 dose (0.4 and 1.03 ml/kg), the difference between ACT with irinotecan vs. irinotecan alone was not significant (p > 0.27 and 0.18 respectively). Overall, increasing the PS101 dose increased the mean normalized body weight loss (p < 0.001) (**Figure 2C**).

### Tumour Volumes

**Figure 3:**
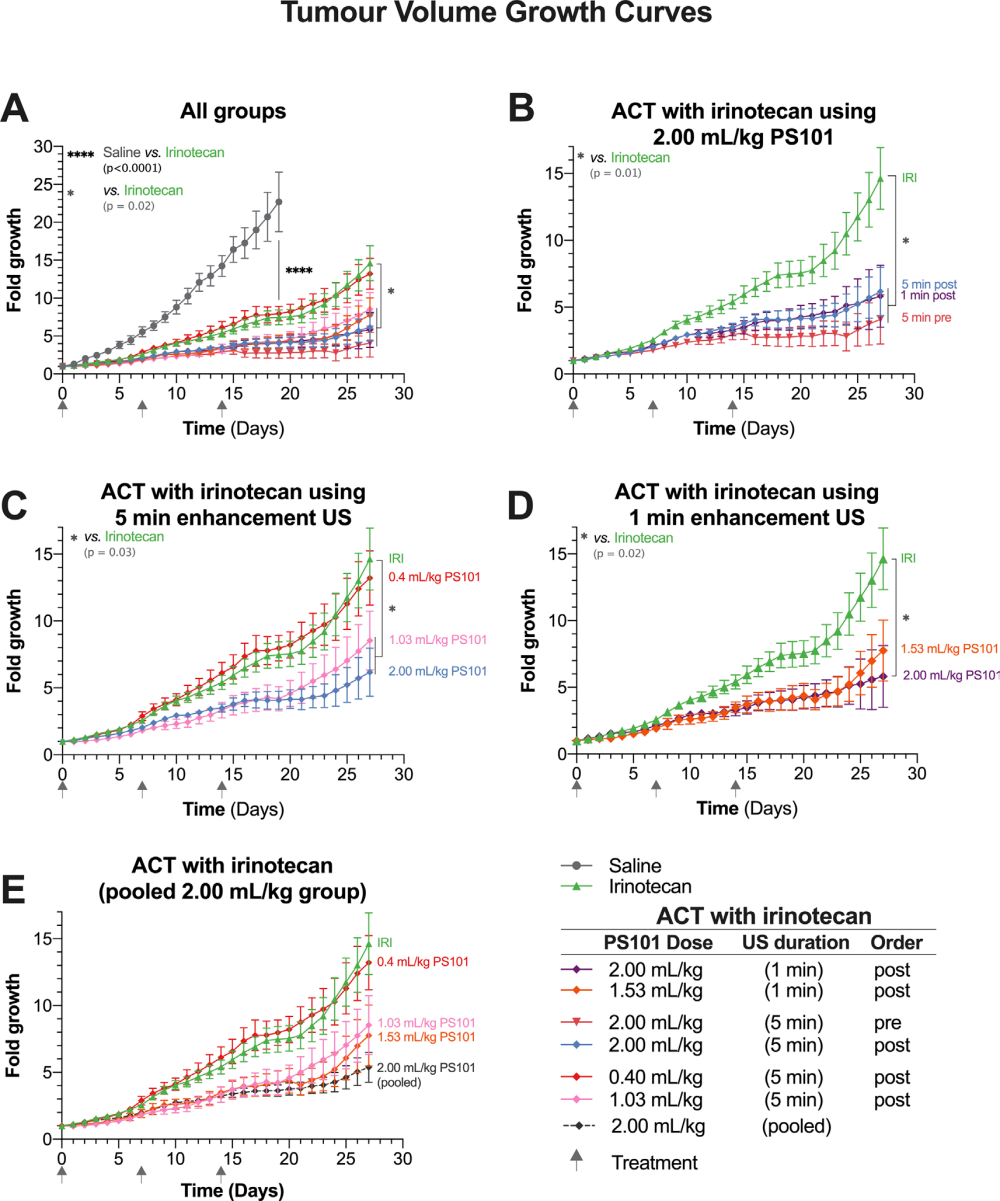
Tumour growth as a function of time. Each panel shows a subset of the data to aid comparisons. (Panel **A**) shows all the groups within this study in a single graph. (Panel **B**) focuses on the groups treated using Acoustic Cluster Therapy (ACT) with irinotecan at 2.00 ml/kg PS101. (Panel **C**) is a “dose response” study and shows the groups treated using ACT with irinotecan where PS101 is administered post irinotecan, with 5 min duration ultrasound (US) at three different doses of PS101. (Panel **D**) compares the efficacy of irinotecan alone vs. ACT with irinotecan where PS101 is administered after irinotecan, with one-minute enhancement US at two different PS101 doses. (Panel **E**) pools all the mice that received 2.00 ml/kg PS101 and compares the data to those for the irinotecan alone and other ACT with irinotecan.

**Figure 3** shows tumour relative volume as a function of time. Irinotecan alone was associated with significant tumour inhibition compared to the saline controls, clearly observable in **Figure 3A** (p < 0.0001, day 19, unpaired t-test). Overall, at day 27, all ACT with irinotecan groups, except for the lower dose of 0.40 ml/kg PS101 (p = 0.641) showed significantly inhibited tumour growth when compared to irinotecan alone (p = 0.002–0.034).

**Figure 3B** compares the groups treated using ACT with irinotecan at a dose of 2.00 ml/kg PS101. There was no difference between the growth curves for 5 and 1 min enhancement-US duration (p = 0.904), in the groups where PS101 and US were administered post-irinotecan. While there was an observable difference between the 5-min enhancement-US duration pre-irinotecan group compared to both post-irinotecan groups, this difference was not significant (p = 0.495 vs. 5 min and 0.555 vs. 1 min). The slight separation between the pre-irinotecan and post-irinotecan groups was observable approximately 2 days after the second treatment (day 9) where the mean tumour volume of the post-groups was approximately 0.5-fold larger than the pre-groups. Two days after the third treatment (day 16) a slight mean tumour volume regression was observed in the pre-irinotecan group only. The separation continued throughout the 27-day period. A violin plot of all the mice that survived to the end of the treatment can be seen in **Supplemental Figure 2A**.

The normalized tumour volumes of the dose-response study in **Figure 3C** shows the groups that received PS101 and enhancement US for 5 min post-irinotecan with various PS101 doses. In general, increasing the dose of PS101 improved the treatment efficacy observed as increased tumour inhibition. As previously stated, the 0.4 ml/kg PS101 group showed no difference to irinotecan alone. At 1.03 ml/kg PS101, the mean tumour volume was 6.1-fold smaller than the irinotecan-alone group at day 27 but the difference was barely significant (p = 0.050). Increasing the PS101 dose to 2.00 ml/kg resulted in a tumour inhibition of 8.5-fold vs. irinotecan alone at day 27, i.e., less than half the tumour volume, and was statistically significant (p = 0.023).

The effect of different PS101 doses when applying enhancement US for 1 min can be seen in **Figure 3D**. In general, there was no difference observed between the two doses of 1.53 ml/kg vs. 2.00 mg/kg PS101. At day 25, a small separation was observed between these two groups, but this is a result of a single mouse with rapid tumour growth 10 days after the last treatment cycle. Hence, only the 2.00 ml/kg group was significantly different from irinotecan alone (p = 0.018).

As no significant difference was observed between the 2.00 ml/kg PS101 groups, independent of the enhancement US duration or order of administration in relation to the irinotecan, all the mice in these groups were pooled and compared to the other PS101 doses in **Figure 3E**. Here, a clear trend can be seen that increasing the PS101 dose resulted in an extended period of tumour inhibition post treatment that monotonically increased with PS101 dose.

### Median Overall Survival

**Figure 4:**
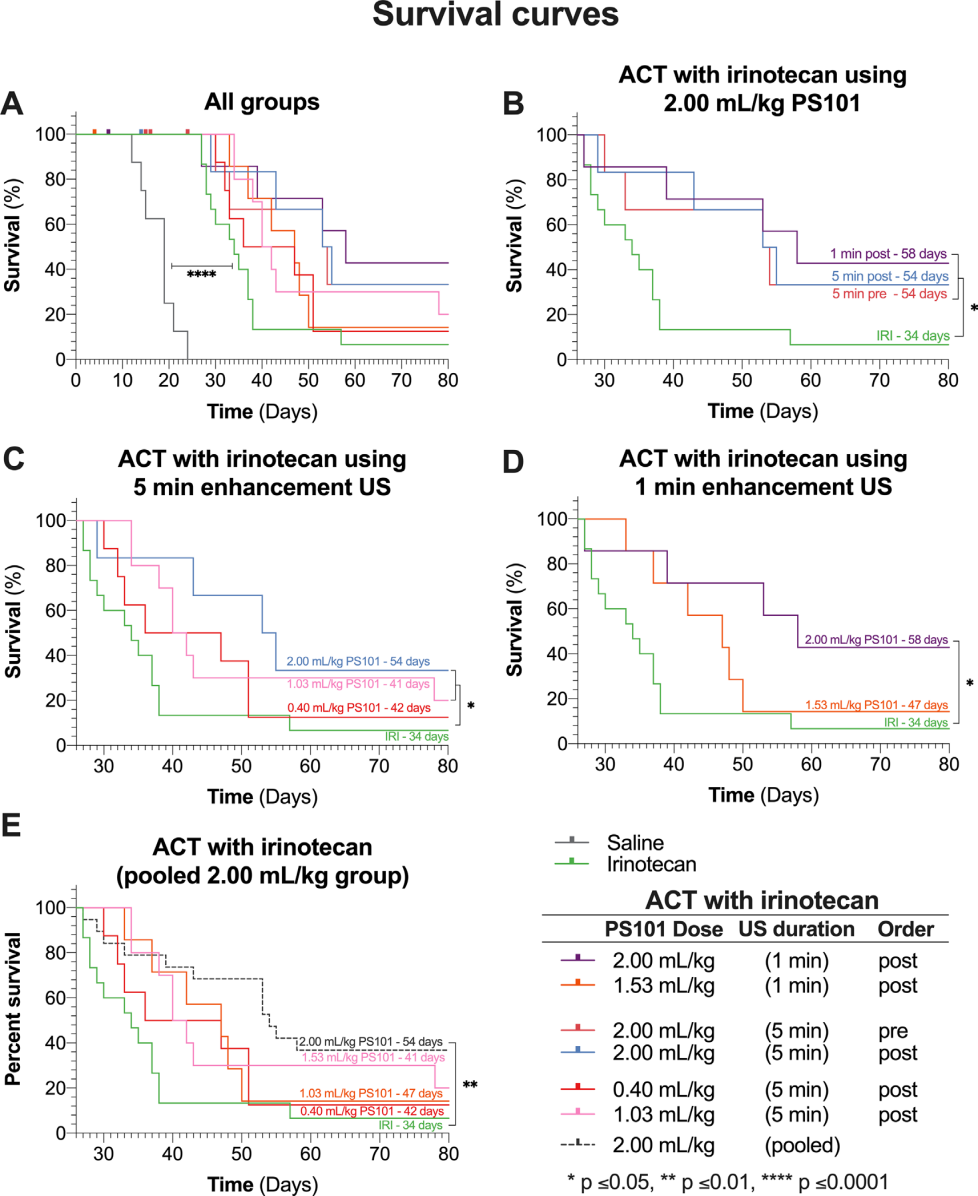
Survival curves of all groups in the study. Each panel shows a subset of the data, each for chosen to allow specific comparisons. The labels on the survival curves indicate the groups and the median survival. (Panel **A**) shows the survival curves of all the groups in the study. Mice that did not complete the full 28 days of treatment are marked as censored subjects. (Panel **B**) compares results for various enhancement-US durations and whether PS101+US was administered pre-irinotecan or post-irinotecan, for mice that received 2.00 ml/kg PS101. (Panel **C**) shows the survival curves of the dose response study for groups that received ACT with irinotecan with 5-min enhancement US at three doses. (Panel **D**) shows the survival curves of mice that received PS101 and US post-irinotecan with one min of US enhancement at two different PS101doses. (Panel **E**) pools all the mice that received 2.00 ml/kg PS101 and compares the survival curve to those for the other ACT with irinotecan groups and irinotecan alone.

**Figure 4** shows the effect of ACT with irinotecan in terms of the overall survival. Mice not treated with any therapeutic, i.e., just saline, had a median survival of 19 days. Treating with irinotecan increased median survival to 34 days and was significant (p < 0.0001). All groups treated using ACT with irinotecan showed an increased median survival (**Figure 4A**).

**Figure 4B** compares the groups treated using ACT with irinotecan at 2.00 ml/kg dose of PS101. There was no significant difference between any of the ACT with irinotecan treated groups. Comparing to irinotecan alone, only the 1 and 5 min post-irinotecan groups were significant, increasing median survival from 34 days to 58 and 54 days (p = 0.016 and 0.050), respectively.

The survival results from the “dose response” study (**Figure 4C**) showed that performing ACT with irinotecan using a PS101 dose of 0.40 ml/kg did not significantly improve survival (p = 0.295) over irinotecan alone due to early dropouts but did increase the median overall survival by 8 days (from 34 to 42 days). Increasing the PS101 dose to 1.03 ml/kg further increased survival rendering it significantly different from that with irinotecan alone (p = 0.027) with a median of 41 days. Further increasing the PS101 dose to 2.00 ml/kg further improved the efficacy of irinotecan resulting in a median survival of 54 days (p = 0.050).

**Figure 4D** compares the survival of the groups that underwent enhancement US for one min at two PS101 doses. One minute of US enhancement post-irinotecan at a PS101 dose of 1.53 ml/kg resulted in a survival increase of 13 days over irinotecan alone, from 34 to 47 days. Nevertheless, this result was not significant (p = 0.115). Increasing the PS101 dose to 2.00 ml/kg resulted in a further improvement in survival to 58 days, rendering it significantly better than irinotecan alone (p = 0.016). This was the longest survival of all the groups in this study.

Once again, as observed in the tumour volume analysis, there was no significant difference in overall survival between all the groups treated using ACT with irinotecan at a PS101 dose of 2.00 ml/kg, independent of the enhancement-US duration and whether PS101+US was administered before or after irinotecan. Hence, the data from these groups were pooled and compared with those for the other PS101 doses (**Figure 4E**). Pooling the data resulted in a median overall survival of 54 days. There was no significant difference between the pooled group and any 2.00 ml/kg group (p = 0.980). Comparing the pooled data to irinotecan alone improves the significance over irinotecan alone (p = 0.002 vs. p = 0.016).

#### Complete Responders

All groups except for the saline group showed complete responders (**Table 3**). In the irinotecan-alone group, while all mice were able to complete the treatment and recovery period, there was only 1 out of 15 mice (7%) that showed complete response. In contrast, all groups treated using ACT with irinotecan showed a higher percentage of responders than the irinotecan alone group (13%–22% vs. 7%). When pooling the 2.00 ml/kg PS101 groups 5 out of 26 mice (19%) showed complete response; i.e., more than a tripling of complete response. However, the increase in complete responders was not significant (p = 0.388).

**Table 3:** Summary of the number of mice that were able to complete the treatment plus recovery period, and the number of complete responders for each group.

Group	Description	Number completed treatment and recovery period	Number of complete responders
1	Saline	0 out of 8 (0%)	0 out of 8 (0%)
2	Irinotecan only	15 out of 15 (100%)	1 out of 15 (7%)
3	0.40 ml/kg PS101, 5 min, post	8 out of 8 (100%)	1 out of 8 (13%)
4	1.03 ml/kg PS101, 5 min, post	10 out of 10 (100%)	2 out of 10 (20%)
5	1.53 ml/kg PS101, 1 min, post	7 out of 8 (88%)	1 out of 8 (13%)
6	2.00 ml/kg PS101, 5 min, post	6 out of 8 (75%)	1 out of 8 (13%)
7	2.00 ml/kg PS101, 1 min, post	7 out of 9 (78%)	2 out of 9(22%)
8	2.00 ml/kg PS101, 5 min, pre	6 out of 9 (67%)	2 out of 9(22%)
	2.00 ml/kg (pooled)	19 out of 26 (73%)	5 out of 26 (19%)

### Dose Response

**Figure 5:**
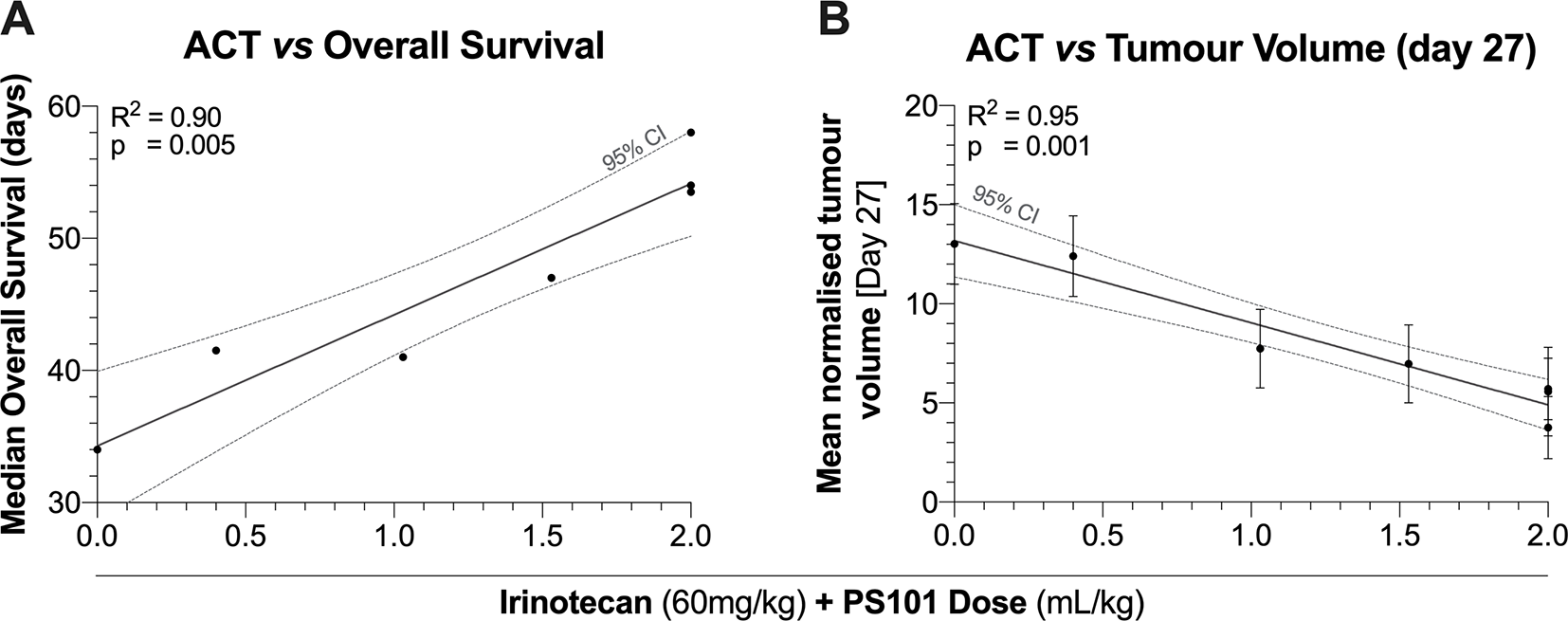
Median overall survival (Panel **A**) and tumour volume (Panel **B**) as a function of the PS101 dose employed when ACT was used to enhance treatment with irinotecan. Increasing the PS101 dose was related to both an increased survival and a reduced tumour volume at day 27.

**Figure 5** shows the correlation between ACT with irinotecan at various doses vs. median overall survival and tumour volume at day 27. In general, an increasing PS101 dose is associated with an increased overall survival and a decreased tumour volume at day 27. The linear regression of PS101 dose on overall survival indicated a slope of 1.46 days/ml/kg of PS101 with a highly significant correlation (95% CI = 0.90 to 2.02, R^2^ = 0.90, p = 0.005) in addition to the 34 days of survival when treated with irinotecan alone (**Figure 5A**).

A similar beneficial trend was observed for tumour volume (**Figure 5B**). The linear regression of PS101 dose on mean normalized tumour volume indicated a slope of -0.68-fold/ml/kg PS101 with a highly significant correlation (95% CI = -0.86 to -0.51, R^2^ = 0.95, p = 0.001). Furthermore, the same trend was observed for the TGI percentage where the slope indicated an inhibition of 32.27%/ml/kg PS101 (95% CI = 20.19 to 44.36, R^2^ = 0.93, p = 0.008, **Supplemental Figure 2B**).

### Liver Toxicity

**Figure 6:**
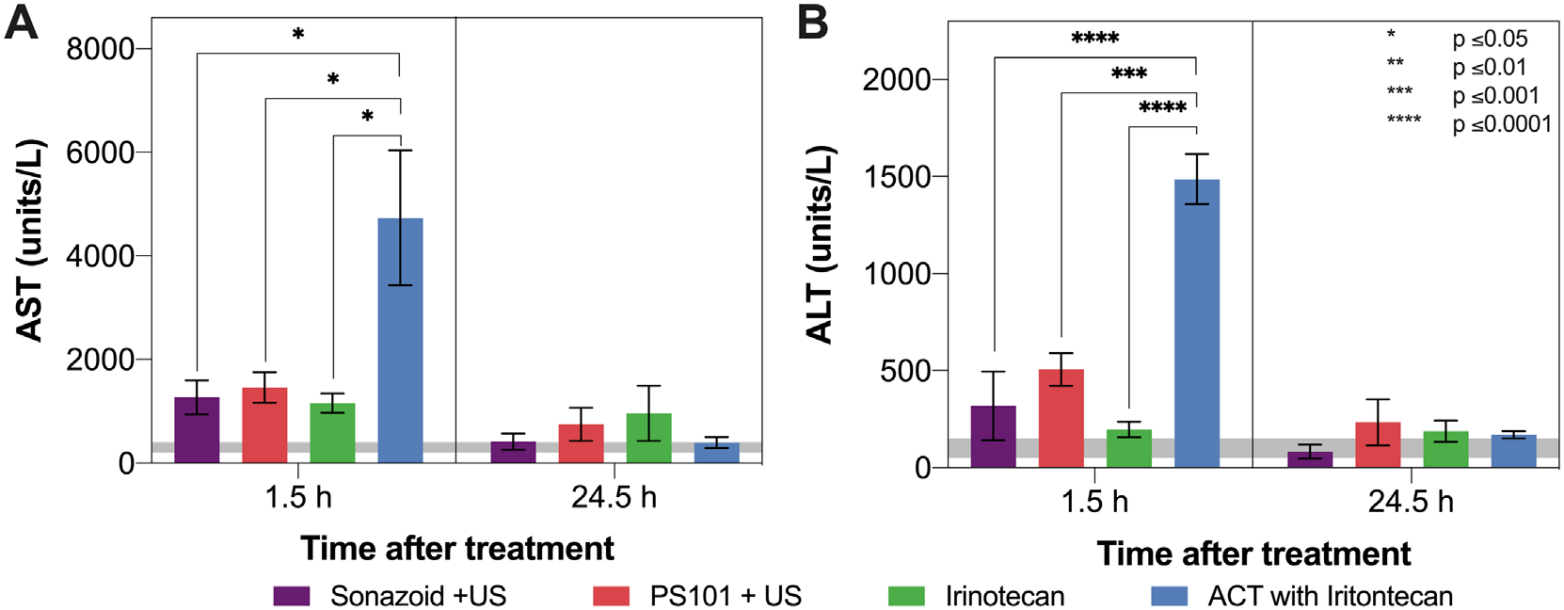
Aspartate aminotransferase (AST) (Panel **A**) and alanine aminotransferase (ALT) (Panel **B**) levels 1.5 and 24.5 h after treatment start. The grey horizontal bar shows normal values reported in literature. Only ACT with irinotecan 1.5 h after treatment start shows a significant difference to any other groups. Decreased levels were observed for all groups after 24.5 h.

**Figure 6** shows the results from the liver toxicity study. In general, 1.5 h after the treatment start all groups exhibited elevated AST and ALT levels when compared to literature normal values. Only the group treated using ACT with irinotecan showed a significant difference, indicating there is a compounding effect when combining ACT with irinotecan (p = 0.0152 – 0.0263 for AST at 1.5 h, p = 0.0004 – < 0.0001 for ALT at 1.5 h). After 24.5 h the mean levels of both enzymes decreased, reaching levels that were not significantly different from normal. At this timepoint, the was no significant difference between any of the groups.

## Discussion

The use of ACT with irinotecan for the treatment of CRC showed a significant improvement in both TGI and overall survival when compare to irinotecan alone. The number of complete responders more than doubled when performing ACT with irinotecan vs. irinotecan alone. Performing the PS101+US before or after the irinotecan injection had no significant effect on the improved efficacy. In addition, applying US for 1 vs. 5 min also resulted in no significant difference. The dose-response study showed that in the doses evaluated there was a linear correlation with therapeutic efficacy.

### Toxicity

On the observed increased level of toxicity (c.f. **Figure 2**), several studies have shown that the gastrointestinal toxicity of irinotecan is influenced by the intestinal microbiota and inflammation conditions (Brandi et al., 2006; Chityala et al., 2017). In addition, treatment with microbubbles has been shown to induce caecal lesions in murine models (Rasmussen et al., 2003) and the development of such is likely to enhance the gastrointestinal toxicity of irinotecan. Furthermore, in the current study, with tumours inoculated on the flank of the animals, collateral US insonation of the intestine was inevitable due to the small size of the animal. It is hypothesised that the increase in irinotecan toxicity observed in this study is due to an increase in inflammatory conditions from the development of caecal lesions and/or to an increased concentration of irinotecan in the intestine, due to collateral insonation. Hence, it is regarded appropriate to have excluded from the survival analysis mice that were unable to complete the treatment and recovery period. While commercial microbubbles and PS101 do have different physical properties, and this may influence the formation of caecal lesions, it is important to note that a large portion of PS101 is indeed a commercial US contrast agent; Sonazoid.

In previous studies, which used ACT at similar PS101 doses to those employed here but with an experimental configuration that shielded the abdomen from the US exposure, no weight loss was observed in any of the groups, including an ACT alone (i.e., PS101+US) group (van Wamel et al., 2016b). Furthermore, extensive toxicity studies have been performed on other species and this phenomenon was not observed (Myhre et al., 2016). This strongly supports the theory that the weight loss observed in this study is due to development of caecal lesions and/or collateral insonation of the intestines resulting in enhanced off-site delivery of irinotecan, and that this was not a systemic toxicity issue.

It should be noted that such effects are very unlikely to translate to the clinical application of ACT for enhancing the efficacy of irinotecan in the treatment of CRC in humans; collateral insonation of the intestine is unlikely in the larger species and the development of caecal lesions upon treatment with microbubbles is specific to murine species (Dirven et al., 2003).

The liver toxicity study showed that PS101+US did not induce any additional toxicity when compared to using the clinical US contrast agent Sonazoid™+US. This indicates that there is no acute or transient liver toxicity due to the positively charged particles in the PS101 formulation. As both the elevated AST and ALT levels were transient and dropped close to normal levels after 24.5 h, this suggests that the mechanism behind ACT may also be transient.

### Growth Inhibition and Survival

As noted from **Figures 3** and **4**, all groups treated using ACT with irinotecan showed a decreased tumour growth rate and an increased median survival compared to irinotecan alone. At the highest PS101 dose investigated a more than 70% reduction in tumour volume was observed vs. drug alone at day 27. Also, a more than 70% increase in median survival vs. drug alone was observed. Even though not statistically significant, the fraction of complete responders in the highest PS101 dose groups was 26% vs. 7% for drug alone. The level of this enhancement effect is comparable to other studies on other disease models treated with different drugs (van Wamel et al., 2016b) further indicating the drug- and disease-agnostic nature of the ACT concept.

Under the assumptions that the generated ACT bubbles have a lifetime of approximately 5 min (van Wamel et al., 2016a) and that the drug-delivery enhancement provided by the biomechanical effects of ACT would be optimal if applied when the drug is actually in the vascular compartment, the procedures applied in the previous studies have all performed the PS101 injection + US procedures after administration of drug, at a time that is close to the drug’s maximum plasma concentration. Surprisingly, in this study, we observed no significant difference between 1 or 5 min of enhancement-US insonation, nor between performing the PS101 injection + US administration before vs. after administration of drug. With regards to the length of US insonation, this finding could indicate that the lifetime of the ACT bubbles in the present work was shorter than that observed in other studies (van Wamel et al., 2016a). Previously, the 5-min lifetime observation was made in the absence of low frequency enhancement-US insonation, which could be postulated to decrease the lifetime. Alternatively, the observation that insonating with enhancement US for longer than 1 min conveys no additional benefit could mean that all of the beneficial effects are induced within the first minute, i.e., they saturate after a short period of US insonation and may occur partly as a consequence of activation-US insonation as well as enhancement-US insonation. This possibility is partially consistent with the observations made in van Wamel et al. (van Wamel et al., 2016a), which reported a significant and substantial improvement of tumour dye-uptake by using ACT with 800CW-PEG dye, vs. dye alone, even when insonating for 45 s with only high-frequency activation-US. Nevertheless, it is important to note that the addition of 5 min of enhancement-US insonation roughly doubled the enhancement of dye delivery in the tumour.

With regards to the sequencing of the procedure, the lack of significant difference between the results for the predrug vs. postdrug application of PS101+ US procedures demonstrated that the drug does not need to be present in the vascular compartment at time of PS101+US administration. This observation would seem to indicate that PS101+US induces an effect on the tumour vasculature and/or interstitial structures that persists and allows for enhanced uptake/distribution of drug even some time after the procedure. This is also consistent with observations made in van Wamel et al (van Wamel et al., 2016a) which reports an increase in uptake of a drug surrogate 1 to 2 h after the PS101+US procedure. This is further corroborated by Åslund et al. (Åslund et al., 2017), which investigated opening of the blood brain barrier with ACT and found that its effect on uptake of gadodiamide in the brain tissue slowly decreased over a period of 72 h, indicating that the microvascular fenestrations that the PS101+US procedure may have opened or induced, close rather slowly. Both these observations indicate the need for further work to fully understand the mechanisms involved but importantly also indicate the noncritical nature of sequencing and timing, which allows for suitable flexibility in clinical applications.

### Dose/Response

As indicated from **Figure 5**, the dose response relation for PS101, evaluated both as a function of tumour size at day 27 and median survival, seems linear over the range investigated, with no sign of saturation effects. This would indicate that PS101 doses higher than 2.00 ml/kg could lead to even stronger enhancement of a drug’s therapeutic benefit. Nevertheless, it should be considered whether such high doses would be feasible in a clinical regime and how a preclinical murine dose would relate to the clinical counterpart.

As the ACT concept is currently in early clinical trials at Royal Marsden Hospital (UK), where it is explored for treatment of hepatic metastases from CRC and PDAC cancers in combination with standard of care chemotherapy (FOLFOX/FOLFIRI for CRC origin and nab-paclitaxel plus gemcitabine for PDAC origin; ClinicalTrials.gov Identifier: NCT04021277), this research provides an important step into understanding the importance of both administration order and dose.

### Limitations and Future Work

While this study has shown that ACT with irinotecan can improve the therapeutic efficacy when compared to irinotecan alone, the mechanism behind this improvement was not investigated. While it may be speculated that this improved efficacy may be due to increased delivery of the drug to the target tissue, this remains to be verified. There are numerous other mechanisms that may affect tumour growth which may be induced by ACT and may be synergistic with the action of the chemotherapeutic agents, such as, changes in intracellular signalling (Furusawa et al., 2012; Haugse et al., 2019), vasculature (Keravnou et al., 2016; Kotopoulis et al., 2017), increased immune response and metabolic activity (Casey et al., 2010). These, and other potential mechanisms, should be investigated in future work to fully understand the biological response to ACT.

## Conclusion

ACT can significantly enhance the inhibition of tumour growth and increase the overall survival benefit provided by irinotecan in a subcutaneous human CRC xenograft. The improved efficacy of ACT with irinotecan was shown to increase linearly with the dose of PS101 in the range investigated (0.40–2.00 ml/kg) for both tumour inhibition and overall survival. There was no significant difference in performing ACT before or after the irinotecan injection. Furthermore, there was no significant difference performing the US enhancement process for 1 or 5 min.

The results from this study indicate the flexibility of ACT, which may have implications for its application in the clinic.

## Data Availability Statement

The datasets generated from this study can be found in the public repository hosted by “LabArchives”. Full URL: https://mynotebook.labarchives.com/share_attachment/My%2520Notebook/MjAuOHw0OTA0MTEvMTYtNC9UcmVlTm9kZS8zNjcxNzgxNjAwfDUyLjg=.

## Ethics Statement

The animal study was reviewed and approved by The ICR Animal Welfare & Ethical Review Body.

## Author Contributions

NB, AH, AS, GB, VK, SKv, PS, and JB contributed to the conception and design of the study. NB, AS, and GB performed the experiments. NB, AH, SKo, SKv, PS, and JB all cowrote the manuscript. All authors contributed to the manuscript revision, read, and approved the submitted manuscript.

## Funding

This research was funded by Phoenix Solutions AS, partially through Research Council of Norway grant no. 228604.

## Conflict of Interest

Authors AH, SKo, SKv, and PS were employed by the company Phoenix Solutions AS at the time of manuscript submission. Authors NB, AH, SKv, and PS are shareholders of Phoenix Solutions AS at the time of submission.

The remaining authors declare that the research was conducted in the absence of any commercial or financial relationships that could be construed as a potential conflict of interest.

The authors declare that this study received funding from Phoenix Solutions AS. The funder had the following involvement with the study: study design, data analysis, decision to publish, and preparation of the manuscript.
