# Supplementary material for: Therapeutic Dose Response of Acoustic Cluster Therapy in Combination With Irinotecan for the Treatment of Human Colon Cancer in Mice
Source: Front Pharmacol. 2019 Nov 19;10:1299. doi: 10.3389/fphar.2019.01299 (PMC6877694; doi:10.3389/fphar.2019.01299)
Supplement: Supplementary file 1 [file Image_1.pdf]

Supplementary Material

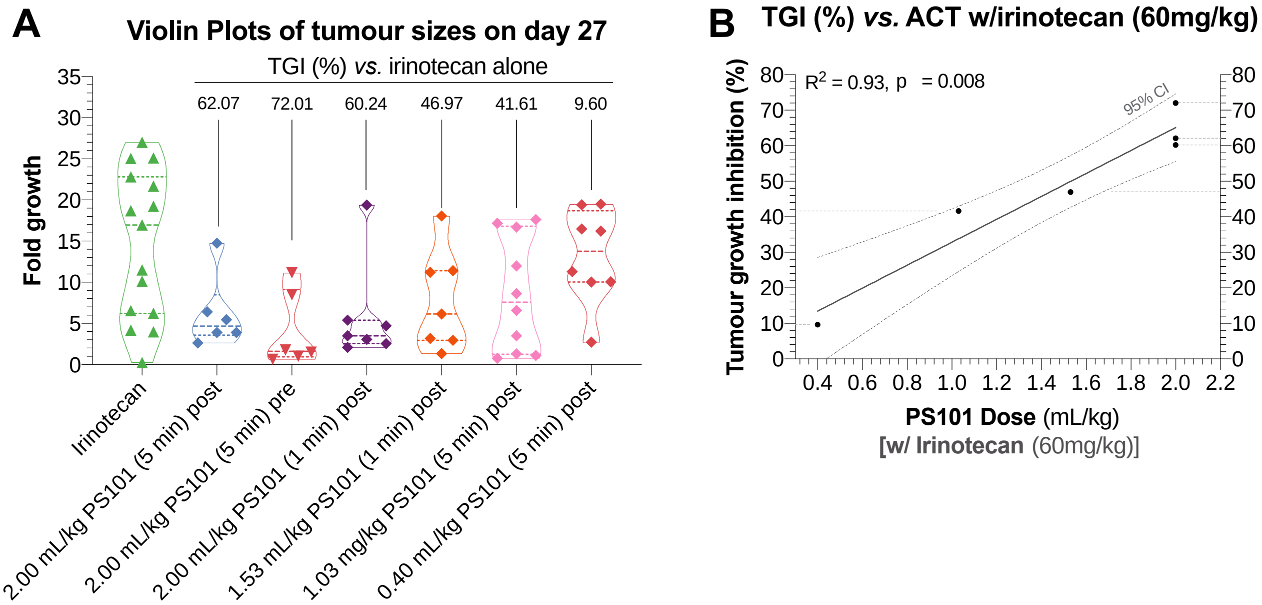

Supplemental Figure 1: Tumour fold growth of all mice at end of treatment and recovery period and correlation to the TGI percentage. **Panel A** shows a violin plot the tumour fold growth of all mice that survived the treatment and recovery period on day 27. The violin plot assists in visualising the bimodal distributions (*e.g.*, irinotecan) and single outliers in the ACT with irinotecan groups. The numbers above each group indicate the TGI as a percentage where the irinotecan group is used as the control. **Panel B** correlates the PS101 dose to the TGI percentage. The TGI percentage had a significant correlation to the PS101 dose (p=0.008).
